# Military Graveyards and Epitaphs in India

**Published:** 1888-10-06

**Authors:** 


### Military Graveyards and Epitaphs in India.—I.

By an Old Indian.

Having always had a leaning towards this kind of literature, I have made it a point for many years back to visit such old graveyards as came in my way, and few men have better opportunities of gratifying this kind of taste than a military medical officer. The results of this research are given below, and if not very novel or striking, they are, at least, authentic. In this fact, indeed, is centred any interest they may possess, for they make no higher pretensions, and some of the particulars given may command sympathy if they do not inspire other feelings. The attention that was called a few years ago, through the press, to the neglected state of our cemeteries in the Crimea had, I believe, the effect of rousing the Government of the day to the necessity of doing something for their repair and preservation. Whether this humble effort of mine will have any similar effect in another place or on a larger field, remains to be seen. Indeed, it is not issued with that view. My object is a narrower one, and I shall be quite content with the honour of its appearance in these pages.

The circumstances under which the extracts given below saw the light are as follows; and, imperfect as they are, I yet prefer leaving them to stand as they originally appeared to diminishing their effect by any attempt at improving them. The tale they tell is a simple one, which requires no new setting, and I have no particular reason for supposing that the situation has undergone any very material change since I saw it. Anyhow, I am not aware of any such change, while I am conscious of having set down nought respecting it in malice, and this being so I will proceed, without further explanation or preface to my destination.

Animated by the spirit indicated above, and having an ulterior professional view which need not be dwelt on here, I availed myself of the march of my regiment from Cawnpore and Futtehgurh, during the cold weather of 1866-7, to Rawul Pindee, to visit the graveyards that occurred *en route*. Several of these were near our camping-grounds, while others were associated with historic names and incidents, and all were fraught to me with messages or meanings of grave import. I approached them, accordingly, with feelings of respect and sympathy; and the substance of my gleanings among the tombs appeared some weeks subsequently, under the heading of "Our Graveyards and Epitaphs," in what was then, and is still, I believe, the leading journal of the North-West.

That sentiment of respect for the memory of the dead which inspires the genius of the poet and lends interest to the creations of the painter, and which was always entertained by the most polished nations of antiquity,* finds, we fear, but little response from the materialism or selfishness of our countrymen in India. Our home is no longer in Hindoostan: we are so anxious to leave the country by the speediest means of conveyance and earliest opportunity, that we are unwilling to devote much time to works of supererogation. The tendency of Englishmen, who, deny it as we may, love to look at things from a utilitarian point of view, tempts us to estimate them according to their intrinsic value, and to set little account by those æsthetic considerations which have so much attraction for other nations. And yet there are few places in which one would more naturally look for a different feeling, for though the tenure of life out here is not so uncertain as it used to be, or as some statisticians would, even now, wish us to believe it is, there are other less selfish reasons why greater attention should be paid to the resting-places of our deceased friends. We can, indeed, rarely afford such costly monuments as adorn our national cemeteries of Newington or Kensal Green or Glasnevin, and the tinsel finery and often fulsome panegyrics of Père la Chaise, would be out of keeping with our ideas of propriety.

"Long palls, drawn hearses, covered steeds"

we cannot command, and the exigencies of time and place often require that we should dispense with even that very moderate measure of ceremonial we are wont to bestow on the sepulture of our dead in more temperate climates. But making all due allowance for these obstacles, we yet think that something better might be done in the way of monuments for our dead, and that the neglect and indifference with which their resting-places are now, in too many instances, treated, call aloud for censure and correction.

We do not allude here to those special memorials to be found in the Presidency towns and more particularly in Calcutta, where, as any casual passenger might have noticed, the "human face divine" of the worthies there commemorated, was, until within a very recent period, often undistinguishable through the favours bestowed on them by crows. We cannot attain to, neither do we covet, such structures as enclose the remains—while they proclaim the pride—of Akbar, Munnoo Begum, Runjeet Singh, or the Wazir of Oude; but there is a medium in all things, and we fear that this has never yet been attained in India. If we except the shaky weatherbeaten obelisk at Chillianwallah, a pillar of red granite from the quarries of Futtehpore Sikree, erected by friends in the public gardens at Agra to the memory of General Adams, and the Lucknow memorial to Sir Henry Lawrence, completed a few months ago, there are few, if any other, mural monuments in Upper India to which one can turn with interest or satisfaction.

The wretched extinguisher-like pyramidal structure dedicated to the memory of General Neil in Huzrutgunge, more resembles a drinking fountain in a country village, the crupper-stone of a roadside inn, or the cairn erected by the piety or superstition of a Highlander, or the rude peasantry of Connemara over the last resting-place of a suicide or a malefactor, than a tribute of admiration to departed greatness. Not much more appropriate, though more pretentious, is the Indian *chef d'œuvre* of Baron Marochetti over the well at Cawnpore, which few appreciate and fewer still understand; and the cross marking the scene of the massacre at Futtehgurh, for which the late Bishop Cotton wrote a suitable inscription, might pass unnoticed, so small is it, in an ordinary English cemetery.

Many of the graveyards attached to cantonments formerly occupied by our troops bear witness, by their jungly appearance, effaced inscriptions, and tottering tombs, to an amount of neglect or indifference on the part of those entrusted with their preservation and care, which it is not agreeable to contemplate under the circumstances. A correspondent, who has lately had an opportunity of personally examining several of these in detail, informs us that in many instances their present condition reflects no credit on the local authorities. At Cawnpore, which formerly enjoyed such an evil repute, the graveyard belonging to the old cantonment at Nawabgunge is so overgrown with weeds, and so full of unsightly decaying monuments, as to be almost impervious to the adventurous survivor, who may strive in vain to identify the grave of a departed friend. At Kurnaul, where the remains of General Anson were deposited for a time, and where still repose the bones of some officers and men of the 3rd Light Dragoons and 3rd Regiment of Foot (the Buffs), the epitaphs on several slabs are quite illegible. A solitary chowkidar, on four rupees a-month, lazily stammers forth a *humko nahin malum* to any interrogatory that is addressed to him, and there is no trace of a footpath or of other recent renovation. At Loodianah, where, among many others, and conspicuous by their magnitude, one never fails to find the stones that covered the mangled bodies of the sixty soldiers of the 50th Regiment, and their families, killed by the falling of a barrack in 1846, many of the inscriptions could not be deciphered, and the place wore a deserted look, though within a few hundred yards of the civil lines. As to Jhelum, which enjoys the honour of a monument erected at the cost of Sir William Gomm and his staff in memory of Colonel Drummond, the rats appear to have it all their own way; there was scarcely any appearance of supervision or repair.

(To be continued.)

* For some proof or particulars in point consult Sir G. C. Lewis's learned "Inquiry into the Credibility of Ancient Roman History," vol. i., p. 182, &c.

